# Naloxone awareness and acquisition: Findings from the 2021‒2022 Canadian Postsecondary Education Alcohol and Drug Use Survey

**DOI:** 10.17269/s41997-025-01034-4

**Published:** 2025-04-28

**Authors:** Jacqueline Burt, Emilia Krzeminska

**Affiliations:** Office of Drug Research and Surveillance, Health Canada, Ottawa, ON Canada

**Keywords:** Naloxone, Antagonists, opioid, Students, Canada, Health knowledge, attitudes, practice, Naloxone, Antagonistes, narcotiques, Étudiants, Canada, Connaissances, attitudes et pratiques en santé

## Abstract

**Objectives:**

This cross-sectional study assessed naloxone awareness, acquisition rates, and reasons for acquisition among postsecondary students in Canada aged 17‒25 years.

**Methods:**

Using data from the 2021‒2022 Canadian Postsecondary Education Alcohol and Drug Use Survey, we conducted descriptive analyses of 31,643 students to characterize naloxone awareness, acquisition, and reasons for acquisition overall and by age, gender, race, international student status, and opioid pain reliever (OPR) use. Using multivariable logistic regression, we assessed the relationship between demographic variables and naloxone awareness and acquisition.

**Results:**

Among postsecondary students in Canada, only 47% had heard of naloxone, and only 5% had acquired it in the past year. Significant predictors of naloxone awareness and acquisition included gender, age, race, international student status, and OPR use. Older students, non-binary students, domestic students, and Indigenous students had higher odds of both naloxone awareness and acquisition. Students who had used OPRs in the past year were less likely to be aware of naloxone (AOR = 0.85, 95% CI: 0.80–0.91). However, among those who were aware, they were more likely to have acquired naloxone (AOR = 1.16, 95% CI: 1.01–1.34) than those who had not used OPRs. Among students who had acquired naloxone in the past year, 97% reported their main reason for obtaining it was for use in emergencies involving other people.

**Conclusion:**

Low naloxone awareness and acquisition among postsecondary students in Canada represent an important public health gap. Increasing naloxone awareness and acquisition may play an important role in enhancing safety on campuses and beyond.

## Résumé

**Objectifs:**

Cette étude transversale a évalué la connaissance de la naloxone, les taux d’acquisition et les raisons de son acquisition parmi les étudiants canadiens de niveau postsecondaire âgés de 17 à 25 ans.

**Méthode:**

En utilisant les données de l’Enquête canadienne sur la consommation d’alcool et de drogue dans les établissements d’enseignement postsecondaire de 2021–2022, nous avons effectué des analyses descriptives sur 31 643 étudiants pour caractériser la connaissance de la naloxone, son acquisition et les raisons de cette acquisition, globalement et en fonction de l’âge, du genre, de la race, du statut d’étudiant international et de la consommation d’opioïdes. À l’aide d’une régression logistique multivariée, nous avons évalué la relation entre les variables démographiques et la connaissance et l’acquisition de la naloxone.

**Résultats:**

Parmi les étudiants de l’enseignement postsecondaire au Canada, seuls 47% d’entre eux avaient entendu parler de la naloxone, et seuls 5% en avaient fait l’acquisition au cours de l’année écoulée. Les indicateurs significatifs de la connaissance et de l’acquisition de la naloxone sont le genre, l’âge, la race, le statut d’étudiant international et la consommation d’opioïdes. Les étudiants plus âgés, les étudiants non-binaires, les étudiants nationaux et les étudiants autochtones avaient des chances plus élevées de connaître la naloxone et d’en avoir déjà acquis. Les étudiants ayant consommé des opioïdes au cours de l’année écoulée étaient moins susceptibles d’être conscients de la naloxone (AOR = 0,85, IC 95%: 0,80–0,91). Cependant, parmi ceux qui en étaient informés, ils étaient plus susceptibles de l’avoir obtenue (AOR = 1,16, IC 95%: 1,01–1,34) que ceux qui n’avaient pas consommé d’opioïdes. Parmi les étudiants ayant acquis de la naloxone au cours de l’année écoulée, 97% ont déclaré que la principale raison pour laquelle ils l’avaient obtenue était pour l’utiliser dans des situations d’urgence impliquant d’autres personnes.

**Conclusion:**

Le faible niveau de connaissance et d’acquisition de la naloxone chez les étudiants de l’enseignement postsecondaire au Canada représente une lacune importante dans les efforts de santé publique. Une meilleure connaissance de la naloxone et son acquisition accrue peuvent jouer un rôle important dans l’amélioration de la sécurité sur les campus et au-delà.

## Introduction

In Canada, opioid use is common among postsecondary students. According to the 2021‒2022 Canadian Postsecondary Education Alcohol and Drug Use Survey (CPADS), 25% of students reported opioid pain reliever (OPR) use in the previous 12 months (Health Canada, 2024a). This is notably higher than the 14% reported in the general Canadian population (Health Canada, 2019). Students are also more likely to use OPRs in a way that puts them at higher risk of harms, such as overdose, by using higher quantities, more often, or for reasons other than prescribed. Among those who had used an OPR in the past year, higher-risk use was reported by 23% of students as compared with 6% of the general population (Health Canada, 2019, 2024a). Although opioids are used primarily to treat pain, postsecondary students also reported using OPRs to help them sleep, feel better, and cope with stress (Health Canada, 2024a).

The use of OPRs among postsecondary students is concerning amid the ongoing opioid crisis. In Canada, 44,592 opioid-related deaths occurred between 2016 and 2023 (Federal, provincial, and territorial Special Advisory Committee on the Epidemic of Opioid Overdoses, 2024). In 2021, 17% of all opioid-related deaths occurred among 20–29-year-olds, the typical age range of most postsecondary students. Opioids were involved in 29% of all deaths within this age group that year (Ledlie et al., 2024). Although the number of overdoses among postsecondary students in Canada is unknown, cases have been reported in the media, fueling discussions of naloxone access on postsecondary campuses (Wyton, 2024). Postsecondary institutions represent an important setting to address naloxone awareness and acquisition.

In Canada, naloxone has reversed thousands of overdoses (Moustaqim-Barrette et al., 2019). This fast-acting opioid antagonist displaces and blocks opioids from brain receptors, reversing symptoms in as little as 2 minutes (Health Canada, 2024b). Despite its wide availability at most pharmacies or local health authorities without a prescription and often free of charge (Health Canada, 2024b), naloxone availability on postsecondary campuses varies by institution (Siebarth, 2017). It is unclear to what extent postsecondary students in Canada are aware of naloxone or acquiring it.

The primary objectives of this study were to assess naloxone awareness and acquisition among postsecondary students in Canada, and to identify key influencing factors. The secondary objective of this study was to describe students’ reasons for acquiring naloxone.

## Methods

### Data and participants

The CPADS is the largest repeat cross-sectional survey on substance use among postsecondary students in Canada. We used data from the 2021–2022 CPADS, collected between November 29, 2021, and April 19, 2022. In that time, 45 schools were recruited across all 10 provinces, representing 23% of the 196 universities, colleges, and CEGEPs (Collège d’enseignement general et professionnel) in Canada. Some schools were not eligible to participate because they did not meet the eligibility criteria. Schools included must: 1) have a registrar office, 2) have more than 500 students, 3) be a not-for-profit public or private school, 4) not offer exclusively online courses, and 5) be non-theological or non-military institutions. Some eligible schools declined, resulting in underrepresentation of British Columbia and no representation in the territories. For students to be eligible, they had to be studying in Canada, online or in-person, and at least 16 years old. In total, 40,931 respondents completed the online survey. An estimated 10.4% of the invited students responded.

To align with Health Canada’s 2021–2022 CPADS results (Health Canada, 2024a), we only included students aged 17 to 25 in our analyses (9288 excluded).

### Study measures

#### Outcome variables

Naloxone awareness was assessed with the question, “Have you heard of naloxone (e.g., Narcan®)?”. Valid response options included “yes” and “no”.

Naloxone acquisition was assessed with the question, “In the past 12 months, have you obtained a naloxone kit?”. Valid response options included “yes” and “no”. Only those who answered “yes” to naloxone awareness saw this question. To report naloxone acquisition for the entire sample of students, we assumed participants who were unaware of naloxone had not obtained a kit.

Reasons for naloxone acquisition were assessed with the question, “What is the main reason you obtained a naloxone kit?”. Valid response options included “in case you need it for yourself”, “in case someone in your family needs it”, “in case a friend needs it”, “in case someone on the street or at a venue needs it”, and “other”. Those selecting “other” could specify a response, which was recoded into existing categories, derived into a new category (“in case anyone needs it”), or excluded if invalid.

#### Predictor variables

Age was assessed with the question, “What is your age?”. Numeric responses of 16 or higher were accepted.

Gender was assessed with the question, “How do you describe yourself?”. Valid response options included “female”, “male”, “transgender female”, “transgender male”, “non-binary gender”, “gender-fluid”, “other gender”. Those selecting “other gender” could specify a response, which was recoded into existing categories or excluded if invalid. Responses were then grouped into three categories: “women” (including “female” and “transgender female”), “men” (including “male” and “transgender male”), and “non-binary” (including “non-binary”, “gender-fluid”, and “other gender”). This approach was taken as the term “non-binary” includes people whose gender identity falls outside the traditional male and female binary, including those who experience both genders at different times or none at all (Matsuno, 2017).

Race was assessed with the question, “What ethnicity are you?”. Valid response options included, “Black”, “East/Southeast Asian”, “Indigenous”, “Latin American”, “Middle Eastern”, “South Asian”, “white”, and “Other”. Those selecting “Other” could specify a response, which was recoded into existing categories or excluded if invalid. Students could select more than one response option. To ensure sufficient numbers for reporting, we combined “Other” and multiracial responses into a single category.

International student status was assessed with the question, “Are you an international student?”. Valid response options included “yes” and “no”.

OPR use was assessed with the question, “Have you ever used any pain relievers?”. Pain relievers were defined as “products that contain opioids such as codeine, morphine or related drugs”. Respondents were asked to exclude drugs such as Regular Tylenol® or Extra Strength Tylenol®, Aspirin®, Advil®, Motrin® or their generic equivalents, and to include prescribed or non-prescribed drugs. Valid response options included “Yes, in the past 30 days”, “Yes, but within the past 12 months”, “Yes, but more than 12 months ago”, and “Never”. We derived a binary variable for OPR use in the past year where “yes” included “Yes, in the past 30 days” and “Yes, but within the past 12 months”, and “no” included “Yes, but more than 12 months ago” and “Never”.

### Statistical analysis

#### Software:

We used RStudio (version 2022.12.0) and the tidyverse, gtsummary, survey, srvyr, and flextable packages for data cleaning, statistical analyses, and exporting (R Core Team, 2022).

#### Weighting: 

In our analyses, we weighted participants by the survey sample weights to ensure the data represented the student population at each school by sex and age and matched the overall student population of the four regions captured in the 2021–2022 CPADS, including Western Canada, Ontario, Quebec, and Atlantic Canada (Health Canada, 2024a).

#### Descriptive analyses:

We calculated percentages and 95% confidence intervals (CI) to characterize the sample and to examine the outcome variables across predictor variables. We suppressed estimates that failed to meet the *Quality Level Guidelines for Weighted Estimates* as defined in the CPADS PUMF User Guide (Health Canada, 2022).

#### Multivariable logistic regressions:

We examined how age, gender, race, international student status, and OPR use relate to naloxone awareness and acquisition among students using multivariable logistic regressions. We reported our results as adjusted odds ratios (AOR) along with 95% CI. We treated age as a continuous variable, and gender, race, international student status, and OPR use as categorical variables. We included age, gender, international student status, and race as predictor variables because they are important determinants of health in this population. These characteristics can cause individuals and certain population groups to experience discrimination, racism, marginalization, or intergenerational trauma, leading to substance use (Health Canada, 2023). We also included OPR use, given people who use opioids are often the focus of harm reduction initiatives like naloxone programs, which can influence naloxone awareness and acquisition.

## Results

### Demographic distribution

After selecting for those aged 17 to 25 years, we included 31,643 students in our analysis. This represents 77% of the 40,931 students who responded to the 2021–2022 CPADS. Table 1 outlines the demographic distribution of the sample. The median age was 21 years old (interquartile range: 19, 23). The sample consisted of 52% women, 46% men, and 3% non-binary students. Many respondents were white (63%) and heterosexual (77%). Most were enrolled full-time (95%) in an undergraduate program (83%). The most common fields of study were arts/humanities/social science (24%) and science/technology (21%). International students comprised 12% of the sample. Most students lived off-campus with family (48%) or off-campus with friends or roommates (32%). One in ten (11%) lived on campus in a school residence.

**Table 1:** Demographic characteristics of postsecondary students in Canada aged 17 to 25 years from the 2021–2022 Canadian Postsecondary Education Alcohol and Drug Use Survey

Characteristic	Unweighted n (weighted %)^a^
Age, years	
17	666 (1.9)
18	4081 (11.5)
19	5596 (16.9)
20	5450 (17.3)
21	4762 (14.6)
22	4013 (12.7)
23	2967 (10.4)
24	2289 (8.0)
25	1819 (6.7)
Age, weighted median (IQR)	21 (19, 23)
Sex	
Male	11,190 (46.1)
Female	20,234 (53.9)
Missing	219
Gender	
Men	11,153 (45.7)
Women	19,333 (51.7)
Non-binary	923 (2.6)
Missing	234
Race	
White	19,529 (62.9)
Black	1031 (3.6)
East/Southeast Asian	2705 (10.7)
Indigenous	325 (1.1)
Latin American	518 (1.9)
Middle Eastern	1001 (3.4)
South Asian	1977 (8.5)
Other race or multiracial	2272 (8.1)
Missing	2285
Sexual orientation	
Bisexual	4893 (14.9)
Gay or lesbian	1327 (4.3)
Heterosexual	22,628 (77.5)
Two spirited	67 (0.2)
Another	1023 (3.1)
Missing	1705
Field of study	
Arts/Humanities/Social Science	7638 (23.6)
Business/Commerce	3339 (11.8)
Education	1326 (4.7)
Engineering	3872 (13.4)
Health Science	4118 (11.9)
Law	890 (2.9)
Medicine	1301 (4.0)
Science/Technology	6761 (20.8)
Other	1991 (6.9)
Missing	407
Level of program	
Advanced degree	3982 (13.8)
Undergrad	25,934 (82.8)
Other	931 (3.5)
Missing	796
Enrollment	
Full-time	29,857 (94.8)
Part-time	1521 (5.2)
Missing	265
International student	
International student	3688 (12.1)
Not an international student	27,663 (87.9)
Missing	292
Residence	
Off-campus alone	2558 (7.8)
Off-campus with family	14,217 (47.9)
Off-campus with friends or roommates	10,353 (31.9)
Other on-campus housing	265 (0.8)
School residence	3802 (11.0)
Unstable housing	36 (0.1^b^)
Other	131 (0.4)
Missing	281
Past 12-month opioid pain reliever use	
Did not use opioid pain relievers	23,028 (75.3)
Used opioid pain relievers	7768 (24.7)
Missing	847

^a^Total unweighted *n* = 31,643

^b^Moderate sampling variability, interpret with caution

### Naloxone awareness

#### Descriptive analysis

A descriptive analysis of naloxone awareness with 95% CI is available in Table 2. Overall, 47% of students had heard of naloxone. By age, awareness ranged from 17% among 17-year-olds to 56% among 25-year-olds. By gender, naloxone awareness was highest among non-binary students (63%), followed by women (52%), and men (40%). By race, the level of naloxone awareness ranged from 26% among Black students to 63% among Indigenous students. Fewer international students had heard of naloxone (13%) as compared with domestic students (52%). Among students who had used opioids in the previous year, 44% were aware of naloxone as compared with 48% among students who had not used OPRs in the previous year.

**Table 2:** Descriptive analysis of naloxone awareness among postsecondary students in Canada aged 17 to 25 years from the 2021–2022 Canadian Postsecondary Education Alcohol and Drug Use Survey

Characteristic	Aware of naloxone Unweighted n (weighted %)	95% CI
Overall	14,907 (46.9)	46.2, 47.5
Age, years		
17	116 (17.2)	14.4, 20.4
18	1463 (34.5)	32.7, 36.2
19	2371 (41.4)	39.8, 42.9
20	2595 (46.0)	44.5, 47.5
21	2462 (51.3)	49.6, 53.0
22	2085 (51.3)	49.5, 53.1
23	1558 (53.0)	50.9, 55.1
24	1259 (54.9)	52.5, 57.3
25	998 (55.6)	52.8, 58.3
Gender		
Men	4224 (39.7)	38.6, 40.7
Women	8923 (52.3)	51.5, 53.2
Non-binary	320 (63.3)	59.5, 66.9
Race		
White	10,388 (54.0)	53.2, 54.9
Black	267 (26.3)	23.2, 29.5
East/Southeast Asian	806 (31.3)	29.4, 33.4
Indigenous	198 (63.0)	56.6, 69.0
Latin American	150 (29.2)	25.0, 33.8
Middle Eastern	281 (27.9)	24.7, 31.4
South Asian	565 (29.0)	26.8, 31.3
Other race or multiracial	1141 (51.9)	49.5, 54.3
International student		
No	14,299 (51.7)	51.0, 52.4
Yes	536 (12.8)	11.6, 14.1
Used opioid pain relievers in past year		
No	11,223 (48.3)	47.5, 49.1
Yes	3443 (44.1)	42.8, 45.4

*CI* Confidence interval

#### Multivariable logistic regression

The results of the multivariable logistic regression for naloxone awareness are available in Table 3.

**Table 3:** Weighted logistic regression results for naloxone awareness among postsecondary students aged 17 to 25 years from the 2021–2022 Canadian Postsecondary Education Alcohol and Drug Use Survey

	Adjusted odds ratio		
Characteristic	OR	95% CI	p-value
Age, years	1.18	1.16, 1.20	< 0.001
Gender			< 0.001
Men	REF	—	—
Women	1.62	1.59, 1.79	< 0.001
Non-binary	2.21	1.92, 2.75	< 0.001
Race			< 0.001
White	REF	—	—
Black	0.45	0.26, 0.36	< 0.001
East/Southeast Asian	0.51	0.34, 0.42	< 0.001
Indigenous	1.40	1.10, 1.90	0.015
Latin American	0.46	0.28, 0.44	< 0.001
Middle Eastern	0.43	0.29, 0.42	< 0.001
South Asian	0.62	0.32, 0.40	< 0.001
Other race or multiracial	0.99	0.83, 1.03	0.910
International student			
No	REF	—	
Yes	0.17	0.15, 0.19	< 0.001
Used opioid pain relievers in past year			< 0.001
No	REF	—	—
Yes	0.85	0.80, 0.91	< 0.001

*OR* Odds ratio, *CI* Confidence interval

#### Age

After adjusting for covariates, older students were more likely to be aware of naloxone than younger students. Each additional year of age was associated with an 18% increase in the odds of awareness (AOR = 1.18, 95% CI: 1.16–1.20).

#### Gender

Compared to men, non-binary students had 121% greater odds of being aware of naloxone (AOR = 2.21, 95% CI: 1.84–2.66) and women had 62% greater odds (AOR = 1.62, 95% CI: 1.52–1.72).

#### Race

Compared to white students, only Indigenous students had higher odds of naloxone awareness (AOR = 1.40, 95% CI: 1.07–1.84). Students of other races had lower odds of being aware of naloxone than white students, up to 55% lower among Middle Eastern students (AOR = 0.43, 95% CI: 0.35–0.51).

#### International student status

Compared to domestic students, international students had 83% lower odds of being aware of naloxone (AOR = 0.17, 95% CI: 0.15–0.19).

#### OPR use

Students who had used OPRs in the past year had 15% lower odds of being aware of naloxone (AOR = 0.85, 95% CI: 0.80–0.91) than students who had not.

### Naloxone acquisition

#### Descriptive analysis

A descriptive analysis of naloxone acquisition with 95% CI is available in Table 4. Overall, 5% of students had acquired naloxone in the past year. By age, naloxone acquisition ranged from 3% among 18-year-olds to 7% among 25-year-olds. Acquisition was highest among non-binary students (11%), followed by women (5%) and men (4%). By race, naloxone acquisition ranged from 2% among South Asian students to 10% among Indigenous students. Fewer international students acquired naloxone (1%) as compared with domestic students (6%). Among students who had used opioids in the previous year, 5.4% had acquired naloxone as compared with 4.8% among students who had not used opioids in the previous year.

**Table 4:** Descriptive analysis of naloxone acquisition among postsecondary students in Canada aged 17 to 25 years from the 2021–2022 Canadian Postsecondary Education Alcohol and Drug Use Survey

Characteristic	Acquired naloxone in past year Unweighted n (weighted %)	95% CI
Overall	(4.9)	4.6, 5.2
Age, years		
17	#	#
18	128 (3.0)	2.5, 3.7
19	208 (3.2)	2.7, 3.8
20	285 (5.1)	4.5, 5.8
21	298 (6.1)	5.3, 7.0
22	242 (6.4)	5.5, 7.4
23	154 (5.1)	4.2, 6.1
24	124 (5.2)	4.3, 6.4
25	116 (7.3)	5.9, 9.0
Gender		
Men	426 (4.1)	3.7, 4.6
Women	1031 (5.4)	5.0, 5.8
Non-binary	97 (10.7)	8.5, 13.5
Race		
White	1135 (5.9)	5.5, 6.3
Black	28 (2.9^a^)	1.8, 4.5
East/Southeast Asian	63 (2.5)	1.8, 3.3
Indigenous	32 (9.5^a^)	6.4, 13.9
Latin American	#	#
Middle Eastern	26 (2.5^a^)	1.6, 4.0
South Asian	40 (1.7^a^)	1.2, 2.5
Other race or multiracial	146 (6.3)	5.3, 7.6
International student		
No	1502 (5.5)	2.1, 9.8
Yes	51 (1.0^a^)	0.7, 1.5
Used opioid pain relievers in past year		
No	1090 (4.8)	4.5, 5.2
Yes	448 (5.4)	4.8, 6.1

*CI *  Confidence interval

^#^Unreleasable due to small sample size or high sampling variability

^a^Moderate sampling variability, interpret with caution

#### Multivariable logistic regression

The results of the multivariable logistic regression for naloxone acquisition are available in Table 5.

**Table 5:** Weighted logistic regression results for naloxone acquisition among postsecondary students aged 17 to 25 years from the 2021–2022 Canadian Postsecondary Education Alcohol and Drug Use Survey

Characteristic	Adjusted odds ratio		
	OR	95% CI	p-value
Age, years	1.13	1.10, 1.16	< 0.001
Gender			< 0.001
Men	REF	—	—
Women	1.25	1.09, 1.44	0.002
Non-binary	2.43	1.81, 3.27	< 0.001
Race			< 0.001
White	REF	—	—
Black	0.62	0.38, 1.02	0.061
East/Southeast Asian	0.49	0.36, 0.67	< 0.001
Indigenous	1.55	1.00, 2.43	0.052
Latin American	0.44	0.21, 0.89	0.022
Middle Eastern	0.52	0.29, 0.65	0.008
South Asian	0.43	0.29, 0.65	< 0.001
Other race or multiracial	1.12	0.91, 1.39	0.292
International student			
No	REF		
Yes	0.25	0.16, 0.40	< 0.001
Used opioid pain relievers in past year			0.031
No	REF	—	—
Yes	1.16	1.01, 1.34	0.031

*OR* Odds ratio, *CI* Confidence interval

#### Age

After adjusting for covariates, older students were more likely to have acquired naloxone than younger students. Each additional year of age was associated with a 13% increase in the odds of acquisition (AOR = 1.13, 95% CI: 1.09–1.15).

#### Gender

Compared to men, non-binary students had 143% greater odds of acquiring naloxone (AOR = 2.43, 95% CI: 1.81–3.27) and women had 25% greater odds (AOR = 1.25, 95% CI: 1.09–1.44).

#### Race

Compared to white students, only Indigenous students had higher odds of naloxone acquisition, though not significantly so (AOR = 1.55, 95% CI: 1.00–2.43). Students of other races had lower odds of having acquired naloxone as compared with white students, up to 57% lower among South Asian students (AOR = 0.43, 95% CI: 0.29–0.65).

#### International student status

International students had 75% lower odds of naloxone acquisition as compared with domestic students (AOR = 0.25, 95% CI: 0.16–0.40).

#### OPR use

Students who had used OPRs in the past year had 16% higher odds of having acquired naloxone than students who had not (AOR = 1.16, 95% CI: 1.01–1.34).

### Reasons for naloxone acquisition

A descriptive analysis of reasons for naloxone acquisition with 95% CI is available in Table 6. We were unable to report reasons for naloxone acquisition by age, gender, race, and OPR use due to high sampling variability. Overall, among students who had acquired a naloxone kit in the past year, 97% reported their main reason for naloxone acquisition was in case someone else needed it. Specifically, most students had acquired naloxone in case someone on the street or at a venue needed it (58%) or in case a friend needed it (24%).

**Table 6:** Descriptive analysis of reasons for naloxone acquisition among postsecondary students in Canada aged 17 to 25 years from the 2021–2022 Canadian Postsecondary Education Alcohol and Drug Use Survey

Variable	Unweighted *n* (weighted%)	95% CI
Main reason for acquiring naloxone		
For someone else	1554 (96.6)	95.3, 97.6
For myself	44 (3.4^a^)	2.4, 4.7
Detailed reason for acquiring naloxone		
In case someone on the street or at a venue needs it	860 (58.4)	55.4, 61.3
In case a friend needs it	421 (24.0)	21.6, 26.6
In case anyone needs it	110 (7.1)	5.7, 8.8
In case I need it for myself	44 (3.4^a^)	2.4, 4.7
In case someone in my family needs it	51 (3.2^a^)	2.3, 4.5
Other	68 (3.9)	2.9, 5.2

*CI* Confidence interval

^a^Moderate sampling variability, interpret with caution

## Discussion

To our knowledge, this was the first study to examine naloxone awareness and acquisition among postsecondary students in Canada. Previous studies occurred early in the opioid crisis and predominantly focused on healthcare providers or small groups of people who use opioids, especially those at higher risk of harm due to factors like injection drug use and street involvement (Goldman-Hasbun et al., 2017; Heavey et al., 2018; Kirane et al., 2016; Nolan, 2017; Tobin et al., 2018). We found that less than half of postsecondary students (47%) were aware of naloxone. Furthermore, only 5% had obtained naloxone in the previous year, predominantly in case of emergencies involving others. We identified age, gender, race, international student status, and OPR use as factors that influenced naloxone awareness and acquisition.

Older students were more aware of naloxone and more likely to have acquired it. This may be due to greater exposure to public health messaging or situations involving naloxone over their lifetime. Additionally, health literacy, which involves obtaining, understanding, and using health information, improves with age in postsecondary students (Kuhn et al., 2021).

In this study, non-binary students and women were more likely than men to have heard of and acquired naloxone. A study of American college students supports these findings, showing that transgender and gender expansive students, followed by women, were more likely to know what naloxone is used for (Freibott, 2024). Overall, very few studies have looked at naloxone awareness and acquisition by gender, including people who are non-binary. To our knowledge, our study is the first to address this gap in the context of postsecondary students in Canada.

We found naloxone awareness and acquisition were highest among Indigenous students. Indigenous populations have been disproportionately affected by the opioid crisis in Canada (Hatt, 2022), potentially leading to greater awareness of harm reduction initiatives. Naloxone awareness and acquisition were also higher among white students than among students of other races. A study on opioid toxicity deaths in Ontario found significant racial disparities in access to healthcare and opioid agonist treatment prior to death (Campbell et al., 2024). Black and Asian individuals were administered naloxone less often than white individuals, suggesting that racialized people face barriers to harm reduction services, which lead to lower naloxone use and overdose prevention training than for white people. The potential barriers discussed by Campbell et al. (2024) could also apply to naloxone awareness and acquisition among racialized postsecondary students, including: cultural stigma and shame around substance use, addiction, and harm reduction; limited diversity among both service users and providers; overt racism within services; and insufficient outreach and representation in service promotion campaigns. In general, more research is needed to understand racial disparities in naloxone awareness and acquisition.

International students had lower odds of both naloxone awareness and acquisition as compared with domestic students. Canada has one of the highest rates of opioid use and overdose globally (Degenhardt et al., 2019), which may contribute to greater awareness among domestic students regarding prevention strategies. Additionally, international students may encounter challenges in navigating the Canadian healthcare system, potentially limiting their access to resources like naloxone. Previous research on mental health service utilization among international students in high-income countries has identified stigma, cultural barriers, and gaps in knowledge as factors that hinder access to these services (Dombou et al., 2023).

Compared to students who had not used OPRs in the past year, students who had used them had slightly lower odds of naloxone awareness, but slightly higher odds of naloxone acquisition. While statistically significant, taking into consideration the CIs of the AORs and the absolute differences in percentages, we believe more research is required to draw definitive conclusions about OPR use as a predictor of naloxone awareness and acquisition among postsecondary students. As we could not find comparable literature in this population, we hope our work provides a baseline for others to draw future comparisons.

A survey of undergraduate students at an American university found that 25% of students had a friend or family member overdose, and 27% witnessed an overdose (Hatteberg et al., 2022). Of those who had a family member or friend overdose, 32% of the overdoses involved opioids. This exposure may explain our finding that of those who acquired naloxone, the vast majority (97%) did so in case someone else needed it. Providing naloxone to those most likely to witness an opioid overdose, including those at risk of an overdose themselves, is in line with recommendations from the World Health Organization (WHO, 2023).

Efforts to increase naloxone availability have grown significantly in Canada since March 2016, when Health Canada changed prescription requirements to enhance public access to naloxone. Pharmacies could distribute naloxone preventively to those at risk of experiencing or witnessing an opioid overdose without individual prescriptions (Health Canada, 2017). This change, along with the introduction of intranasal naloxone, increased naloxone dispensing rates (Antoniou et al., 2021). However, there remains significant potential to improve naloxone acquisition. In our study, we found that 5% of postsecondary students had obtained naloxone in the past year. This was slightly higher than the 2019 Canadian Alcohol and Drug Survey which reported that 2% of Canadians aged 15 years and older had obtained a naloxone kit in the past year (Health Canada, 2019). In the 2017 Survey on Opioid Awareness, only 7% of Canadians aged 18 years and older said they would know how to both obtain and administer naloxone (Statistics Canada, 2018). In March 2024, the co-chairs of the Special Advisory Committee on the Epidemic of Opioid Overdoses, including Canada’s Chief Public Health Officer, advised that carrying naloxone is a step that can be taken by all Canadians to help combat the crisis and save lives (Public Health Agency of Canada, 2024). A recent cost-effectiveness study suggested that distributing naloxone to all Canadians every 3 years would prevent an additional 151 overdose deaths per 10,000 people (Cid et al., 2024). With the growing call for the public to carry naloxone, our findings provide a baseline for future efforts to increase naloxone awareness and acquisition among postsecondary students.

### Strengths and limitations

The CPADS is Canada’s most extensive survey of substance use among postsecondary students, with participation from over 40,000 students across all 10 provinces in 2021–2022. Online administration ensures anonymity, which may encourage more honest responses, increases convenience for respondents, and removes geographical obstacles to participation for students in rural and remote areas.

There are limitations to the study. Postsecondary schools in the territories declined to participate in the 2021–2022 CPADS, and there was underrepresentation in British Columbia, limiting the representativeness of the data and the generalizability of our results to the overall postsecondary student population in Canada. Data are also self-reported and could be subject to recall bias and data entry errors. Finally, the CPADS sample may be skewed towards respondents who are more interested or knowledgeable on substance use given the topic is displayed prominently in the survey’s title. Given these limitations, under- or overreporting of substance use-related behaviours could have resulted in the data (Health Canada, 2024a).

## Conclusion

Our study is the first to examine naloxone awareness, naloxone acquisition, and reasons for acquisition among postsecondary students in Canada. These findings will inform naloxone education programs for students. Increasing naloxone awareness and acquisition may play an important role in enhancing safety on campuses and beyond, potentially contributing to the broader effort to curb the opioid crisis in Canada.

## Contributions to knowledge

What does this study add to existing knowledge?

- This study provides estimates of the prevalence of naloxone awareness and acquisition among postsecondary students in Canada.
- This study identifies factors that influence naloxone awareness and acquisition.
- This study reports the reasons why postsecondary students acquire naloxone.

What are the key implications for public health interventions, practice, or policy?

- Increasing naloxone awareness and acquisition among postsecondary students could be beneficial for public health efforts.
- Understanding factors that impact naloxone awareness and acquisition can help address disparities in these areas.

## Data Availability

A public use microdata file (PUMF) for the 2021‒2022 cycle of the Canadian Postsecondary Education Alcohol and Drug Use Survey is available on the Government of Canada’s Open Government (https://open.canada.ca/data/en/dataset/736fa9b2-62e4-4e31-aea4-51869605b363) portal (Health Canada, 2022). Due to disclosure control measures implemented during the PUMF’s creation, estimates obtained from the PUMF may slightly differ from those in our analysis, which used master data files.

## Code availability

Please contact the corresponding author.

## References

[CR1] Antoniou, T., Martins, D., Campbell, T., Tadrous, M., Munro, C., Leece, P., Mamdani, M., Juurlink, D. N., & Gomes, T. (2021). Impact of policy changes on the provision of naloxone by pharmacies in Ontario, Canada: A population-based time-series analysis. *Addiction,**116*(6), 1514–1520. 10.1111/add.15324 33207025 10.1111/add.15324 PMC8247272

[CR2] Campbell, T. J., Kitchen, S. A., Tadrous, M., Damba, C., Johnson, C. H., Smoke, A., Crichlow, F., & Gomes, T. (2024). Varying circumstances surrounding opioid toxicity deaths across ethno-racial groups in Ontario, Canada: A population-based descriptive cross-sectional study. *BMJ Public Health,**2*, e000480. 10.1136/bmjph-2023-000480 40018176 10.1136/bmjph-2023-000480 PMC11812798

[CR3] Cid, A., Mahajan, N., Wong, W. W. L., Beazely, M., & Grindrod, K. A. (2024). An economic evaluation of community pharmacy-dispensed naloxone in Canada. *Canadian Pharmacists Journal,**157*(2), 84–94. 10.1177/17151635241228241 38463179 10.1177/17151635241228241 PMC10924576

[CR4] Degenhardt, L., Grebely, J., Stone, J., Hickman, M., Vickerman, P., Marshall, B. D. L., Bruneau, J., Altice, F. L., Henderson, G., Rahimi-Movaghar, A., & Larney, S. (2019). Global patterns of opioid use and dependence: Harms to populations, interventions, and future action. *Lancet (London, England),**394*(10208), 1560–1579. 10.1016/S0140-6736(19)32229-9 31657732 10.1016/S0140-6736(19)32229-9 PMC7068135

[CR5] Dombou, C., Omonaiye, O., Fraser, S., Cénat, J. M., Fournier, K., & Yaya, S. (2023). Barriers and facilitators associated with the use of mental health services among immigrant students in high-income countries: A systematic scoping review. *PLoS ONE,**18*(6), e0287162. 10.1371/journal.pone.0287162 37384726 10.1371/journal.pone.0287162 PMC10310021

[CR6] Federal, provincial, and territorial Special Advisory Committee on the Epidemic of Opioid Overdoses. (2024). Opioid- and Stimulant-related Harms in Canada. Public Health Agency of Canada. Retrieved July 3, 2024 from https://health-infobase.canada.ca/substance-related-harms/opioids-stimulants/

[CR7] Freibott, C. E., Vest, N., Stein, M. D., & Lipson, S. K. (2024). Opioid Overdose Knowledge Among Adolescents and Young Adults. *JAMA Pediatrics,**178*(6), 618–620. 38648053 10.1001/jamapediatrics.2024.0561 PMC11036307

[CR8] Goldman-Hasbun, J., DeBeck, K., Buxton, J. A., Nosova, E., Wood, E., & Kerr, T. (2017). Knowledge and possession of take-home naloxone kits among street-involved youth in a Canadian setting: A cohort study. *Harm Reduction Journal,**14*(1), 79. 10.1186/s12954-017-0206-6 29273031 10.1186/s12954-017-0206-6 PMC5741899

[CR9] Hatt, L. (2022). The opioid crisis in Canada (HillStudies) (Publication No. 2021–23-E). Ottawa, ON. Library of Parliament. Retrieved July 25, 2024 from https://lop.parl.ca/sites/PublicWebsite/default/en_CA/ResearchPublications/202123E

[CR10] Hatteberg, S. J., Kollath-Cattano, C., Weller, D. S., 2nd., & Scully, A. E. (2022). Encountering overdose: Examining the contexts and correlates of US college students’ overdose experiences. *Substance Use & Misuse,**57*(10), 1599–1607. 10.1080/10826084.2022.2102188 35877471 10.1080/10826084.2022.2102188

[CR11] Health Canada. (2017). Questions and answers - naloxone. Retrieved June 25, 2024 from https://www.canada.ca/en/health-canada/services/drugs-health-products/drug-products/prescription-drug-list/questions-answers-naloxone.html

[CR12] Health Canada. (2019). Canadian Alcohol and Drugs Survey (CADS): summary of results for 2019. Retrieved June 18, 2024 from https://www.canada.ca/en/health-canada/services/canadian-alcohol-drugs-survey/2019-summary.html

[CR13] Health Canada. (2022). Canadian Postsecondary Education Alcohol and Drug Use Survey (CPADS) 2021-2022 Public Use Microdata Files (PUMF). Retrieved May 28, 2024 from https://open.canada.ca/data/en/dataset/736fa9b2-62e4-4e31-aea4-51869605b363

[CR14] Health Canada. (2023). The Canadian drugs and substances strategy: The Government of Canada’s approach to substance use related harms and the overdose crisis. Ottawa, ON: Government of Canada. Retrieved July 3, 2024 from https://www.canada.ca/en/health-canada/services/publications/healthy-living/canadian-drugs-substances-strategy-approach-related-harms-overdose-crisis.html

[CR16] Health Canada. (2024a). *Canadian Postsecondary Education Alcohol and Drug Use Survey*, 2021–2022: Summary. Retrieved June 18, 2024 from https://health-infobase.canada.ca/alcohol/cpads/

[CR15] Health Canada. (2024b). Naloxone. Retrieved June 18, 2024 from https://www.canada.ca/en/health-canada/services/opioids/naloxone.html

[CR17] Heavey, S. C., Chang, Y. P., Vest, B. M., Collins, R. L., Wieczorek, W., & Homish, G. G. (2018). “I have it just in case” - Naloxone access and changes in opioid use behaviours. *International Journal of Drug Policy,**51*, 27–35. 10.1016/j.drugpo.2017.09.015 29156400 10.1016/j.drugpo.2017.09.015 PMC8934173

[CR18] Kirane, H., Ketteringham, M., Bereket, S., Dima, R., Basta, A., Mendoza, S., & Hansen, H. (2016). Awareness and attitudes toward intranasal naloxone rescue for opioid overdose prevention. *Journal of Substance Abuse Treatment,**69*, 44–49. 10.1016/j.jsat.2016.07.005 27568509 10.1016/j.jsat.2016.07.005

[CR19] Kuhn, L., Bachert, P., Hildebrand, C., Kunkel, J., Reitermayer, J., Wasche, H., & Woll, A. (2021). Health literacy among university students: A systematic review of cross-sectional studies. *Frontiers in Public Health,**9*, 680999. 10.3389/fpubh.2021.680999 35127605 10.3389/fpubh.2021.680999 PMC8814326

[CR20] Ledlie, S., Juurlink, D. N., Tadrous, M., Mamdani, M., Paterson, J. M., & Gomes, T. (2024). Opioid-related deaths between 2019 and 2021 across 9 Canadian provinces and territories. *CMAJ,**196*(14), E469–E476. 10.1503/cmaj.231339 38621782 10.1503/cmaj.231339 PMC11019600

[CR21] Matsuno, E., & Budge, S. L. (2017). Non-binary/genderqueer identities: A critical review of the literature. *Current Sexual Health Reports,**9*, 116–120. 10.1007/s11930-017-0111-8

[CR22] Moustaqim-Barrette, A., Elton-Marshall, T., Leece, P., Morissette, C., Rittenbach, K., & Buxton, J. A. (2019). Environmental scan naloxone access and distribution in Canada. Retrieved July 3, 2024 from 10.14288/1.0379400

[CR23] Nolan, S., Buxton, J., Dobrer, S., Dong, H., Hayashi, K., Milloy, M. J., Kerr, T., Montaner, J., & Wood, E. (2017). Awareness, possession, and use of take-home naloxone among illicit drug users, Vancouver, British Columbia, 2014–2015. *Public Health Reports,**132*(5), 563–569. 10.1177/0033354917717230 28750193 10.1177/0033354917717230 PMC5593231

[CR24] Public Health Agency of Canada. (2024). Joint statement from the co-chairs of the Special Advisory Committee on the Epidemic of Opioid Overdoses – Latest national data on substance-related harms. Retrieved July 3, 2024 from https://www.canada.ca/en/public-health/news/2024/03/joint-statement-from-the-co-chairs-of-the-special-advisory-committee-on-the-epidemic-of-opioid-overdoses--latest-national-data-on-substance-related.html

[CR25] R Core Team. (2022). R: A language and environment for statistical computing. In R Foundation for Statistical Computing. https://www.R-project.org/

[CR26] Siebarth, T. (2017). Universities come to grips with Canada’s opioid overdose crisis. *University Affairs*. Retrieved July 3, 2024 from https://universityaffairs.ca/news/news-article/universities-come-grips-canadas-opioid-overdose-crisis/

[CR27] Statistics Canada. (2018). Results of the Survey on Opioid Awareness, November 2017. Government of Canada. Retrieved June 25, 2024 from https://www150.statcan.gc.ca/n1/daily-quotidien/180109/dq180109a-eng.htm

[CR28] Tobin, K., Clyde, C., Davey-Rothwell, M., & Latkin, C. (2018). Awareness and access to naloxone necessary but not sufficient: Examining gaps in the naloxone cascade. *The International Journal on Drug Policy,**59*, 94–97. 10.1016/j.drugpo.2018.07.003 30075401 10.1016/j.drugpo.2018.07.003 PMC6341469

[CR29] World Health Organization. (2023). Opioid overdose. Retrieved June 24, 2024 from https://www.who.int/news-room/fact-sheets/detail/opioid-overdose

[CR30] Wyton, M. (2024). Coroner’s inquest to probe overdose death of UVic student. *CBC News*. Retrieved June 25, 2024 from https://www.cbc.ca/news/canada/british-columbia/coroners-inquest-sidney-mcintyre-starko-overdose-death-fentanyl-university-victoria-bc-1.7210432

